# Innovations in conditioning and post-transplant maintenance in AML: genomically informed revelations on the graft-versus-leukemia effect

**DOI:** 10.3389/fimmu.2024.1359113

**Published:** 2024-03-20

**Authors:** H. Moses Murdock, Vincent T. Ho, Jacqueline S. Garcia

**Affiliations:** ^1^ Department of Medical Oncology, Dana-Farber Cancer Institute, Boston, MA, United States; ^2^ Bone Marrow Transplant Program, Department of Medical Oncology, Dana-Farber Cancer Institute, Boston, MA, United States

**Keywords:** AML, allogeneic stem cell transplant, graft-versus-leukemia, maintenance, targeted therapy, MRD, genetics

## Abstract

Acute Myeloid Leukemia (AML) is the prototype of cancer genomics as it was the first published cancer genome. Large-scale next generation/massively parallel sequencing efforts have identified recurrent alterations that inform prognosis and have guided the development of targeted therapies. Despite changes in the frontline and relapsed standard of care stemming from the success of small molecules targeting FLT3, IDH1/2, and apoptotic pathways, allogeneic stem cell transplantation (alloHSCT) and the resulting graft-*versus*-leukemia (GVL) effect remains the only curative path for most patients. Advances in conditioning regimens, graft-vs-host disease prophylaxis, anti-infective agents, and supportive care have made this modality feasible, reducing transplant related mortality even among patients with advanced age or medical comorbidities. As such, relapse has emerged now as the most common cause of transplant failure. Relapse may occur after alloHSCT because residual disease clones persist after transplant, and develop immune escape from GVL, or such clones may proliferate rapidly early after alloHSCT, and outpace donor immune reconstitution, leading to relapse before any GVL effect could set in. To address this issue, genomically informed therapies are increasingly being incorporated into pre-transplant conditioning, or as post-transplant maintenance or pre-emptive therapy in the setting of mixed/falling donor chimerism or persistent detectable measurable residual disease (MRD). There is an urgent need to better understand how these emerging therapies modulate the two sides of the GVHD vs. GVL coin: 1) how molecularly or immunologically targeted therapies affect engraftment, GVHD potential, and function of the donor graft and 2) how these therapies affect the immunogenicity and sensitivity of leukemic clones to the GVL effect. By maximizing the synergistic action of molecularly targeted agents, immunomodulating agents, conventional chemotherapy, and the GVL effect, there is hope for improving outcomes for patients with this often-devastating disease.

## Introduction

Acute Myeloid Leukemia (AML) is an aggressive malignancy characterized by hematopoietic stem cell and early myeloid progenitor developmental arrest and aberrant proliferation. Scientific advances over the past two decades have revealed that recurrent leukemia-associated mutations and cytogenetic abnormalities drive disease biology. With the advent of widely available massively parallel (“next generation”) sequencing (NGS) in the research and clinical setting, there is a growing understanding of how patterns of co-mutation inform prognosis and treatment selection at various treatment stages. Allogeneic stem cell transplantation (alloHSCT) is a cornerstone and standard treatment modality for patients with intermediate or higher risk AML in first complete remission (CR1) or beyond, and intensive research efforts have elucidated how the genomic profile of AML influences prognosis in this setting. Additionally, molecularly targeted therapies initially studied and approved in the front-line setting are increasingly being considered as maintenance or pre-emptive therapy in the post-transplantation period to mitigate ongoing risk of relapse, particularly in ultra-high risk genomic subgroups. In this review, we aim to provide an overview of our understanding of how targeted therapies intersect with the graft-versus-leukemia (GVL) effect after alloHSCT, which has long been considered the “modus operandi” of this often lifesaving intervention.

## Genomics and prognosis

Since the publication of the first cancer genome, a cytogenetically normal AML, there has been ongoing interest in understanding the landscape of recurrent driver mutations in AML in order to inform prognosis (1). Prior to this, our understanding of the impact of genetic alterations on treatment outcomes was based on analysis of karyotypes, which we now know lacks the resolution to capture the full scope of pathogenic DNA-level alterations in AML (2–4). Initially, driver gene mutations were investigated in cytogenetically normal cases (5), with subsequent efforts focused on whole-genome and whole exome analysis in a broader set of 200 *de novo* AML cases (6). There was also a growing understanding of how cytogenetics and targeted sequencing approaches could be used in tandem to inform prognosis in clinical trial settings (7). Increasingly large datasets allowed for a more comprehensive assessment of gene mutation frequencies, co-mutation patterns, and development of distinct molecular subgroups that differ based on prognosis and leukemogenic pathways. A seminal example of this approach was the publication by Papaemmanuil and colleagues, who performed comprehensive diagnostic cytogenetic and molecular profiling in 1,540 patients who were enrolled in prospective trials of intensive therapy (8). By assessing driver mutations in 111 cancer genes, they found that 96% of patients had at least one driver mutation in one of 77 loci. Six genes (*FLT3, NPM1, DNMT3A, NRAS, CEBPA*, and *TET2*) were mutated in >10% of the cohort.

Incorporation of AML mutational profiles has led to the adoption of molecularly defined AML subgroups in the latest World Health Organization and International Consensus Classification of myeloid neoplasms classifications (9, 10) and continues to serve as the basis of the AML European Leukemia Net (ELN) risk stratification scheme (11). Most recently updated in 2022, high risk disease is characterized by mutations in *TP53*, secondary-ontogeny associated mutations, and a variety of high risk cytogenetic findings including complex and monosomal karyotypes which have long been known to portend dismal prognosis (12–15). In contrast, favorable risk is defined by mutated *NPM1* without concomitant *FLT3*-ITD mutation, bZIP in-frame mutated *CEBPA* as well as core-binding factor leukemias (16–20). These risk groups are a useful simplification of highly complex effects of co-mutation, clonal hierarchy, and leukemic evolution. The outcomes of patients within these risk groups are variable, modified by co-mutations, baseline clinical variables, and emerging treatment strategies. Examples abound, including the impact of *KIT* mutations and secondary genetic lesions in core-binding factor leukemia, as well as the influence of age on *FLT3*-ITD mutated leukemia (21–23).

## Measurable residual disease

Diagnostic mutations are known to impact outcomes in myeloid malignancies after alloHSCT, which is the preferred consolidative strategy in higher risk disease (24–27). Serial assessment of disease burden using measurable residual disease (MRD) testing over the course of therapy is now an established strategy in AML (28). MRD assessments may be performed using flow cytometric, RT-PCR, or NGS technologies, with the latter genetically focused technologies allowing for MRD assessments in a broader subset of AML cases (29). Molecular MRD has been evaluated for its prognostic utility in the post-up front therapy (30–37), pre-transplant (38–45), post-transplant (46, 47), and relapse/refractory settings (48), but NGS-MRD is not yet ready for widespread clinical adoption. Increasingly, MRD assessments are being studied to guide therapeutic decisions (49–54). We refer the reader to a recent review of MRD in acute myeloid leukemia for a comprehensive discussion on this topic (55).

## Genomics and up-front treatment

Until recently, initial treatment for adults with AML consisted of intensive induction chemotherapy for fit patients and non-intensive therapy with DNA methyltransferase inhibitors (DNMTi, interchangeably referred to as hypomethylating agents [HMA]) for frail or elderly individuals. Intensive induction commonly consists of genomics-agnostic cytotoxic therapies such as the 7 + 3 regimen of an anthracycline and infusional cytarabine or FLAG (fludarabine, cytarabine, and G-CSF) based regimens. Non-intensive therapies have historically also been deployed in a genomics-agnostic manner and include the DNMTi azacitidine and decitabine.

Modern AML therapies (i.e., gilteritinib, ivosidenib with or without DNMTi, enasidenib, DNMTi/venetoclax and low-dose cytarabine/venetoclax combinations) with disease modifying capabilities have altered the prognostic implications of cytogenetics and genetics determined at the time of diagnosis, suggesting dynamic reassessment may be required in the context of therapy. The front-line treatment of older patients with AML has been revolutionized with the introduction of the *BCL2* inhibitor venetoclax, which improved survival outcomes when combined with azacitidine in this patient population in the phase III VIALE-A trial (56). Just as the introduction of FLT3 inhibitors led to evolution in our understanding of the prognostic significance of mutation burden (in the form of allelic ratios) in *FLT3*-ITD, so too venetoclax may be altering the implications of splicing factor (secondary ontogeny-defining) mutations, which are considered adverse risk by 2022 ELN (16, 57).

## Allogeneic stem cell transplantation

Outcomes in patients who relapse after alloHSCT are dismal. In a study of patients with acute nonlymphocytic leukemia relapsing after marrow transplantation, median disease-free survival (DFS) was 6 months (58). In a study of adults with relapsed AML after reduced intensity conditioning (RIC) alloHSCT, 2-year overall survival (OS) from relapse was 14% (59). In a study of MDS and secondary AML, median OS from relapse was 4.7 months with 2-year survival of 17.7% (60). Given these disappointing statistics, there has been great interest in identifying relapse-mitigating strategies. These strategies must consider the genomic profile of the leukemia as well as the complex immunologic landscape which includes the impact of any therapy on the donor immune system, the residual host immune system, and the immune microenvironment. In the remainder of this review, we will discuss innovations in conditioning regimens and post-transplant maintenance primarily through evidence from prospective studies. We will review whether the data suggest these therapies provide post-transplant disease control by cytoreduction or through GVL induction. Post-transplant pre-emptive or prophylactic cytoreductive therapy still has value if it can control MRD clone expansion in the early post-transplant period without causing excess toxicity or interfere with engraftment until the benefits of transplant GVL manifest. A long-standing goal in the alloHSCT field has been to decouple the beneficial anti-leukemic GVL effect after transplant from allo-immunity against non-leukemic tissue, or graft-vs-host disease (GVHD), since presence of GVHD, especially cGVHD, is almost universally associated with lower relapse rates, and even among patients who relapse after alloHSCT, durable remissions are rarely observed unless they subsequently develop GVHD (61). We will also review the emerging scientific data on these agents through a genomic and immunologic lens. Lastly, we will also highlight our limited, but evolving, understanding of how these relapse-prevention strategies impact the GVL effect.

### Genomics and conditioning chemotherapy intensity

Intensive myeloablative conditioning (MAC) regimens offer the benefits of additional leukemic cytoreduction, and faster donor engraftment after transplant by creating more “marrow space” for engrafting cells. However, the potential relapse/engraftment benefit of MAC may be counterbalanced by an increase in regimen related toxicity and transplant-related mortality (TRM, also known as “non-relapse mortality, “NRM”). Scott and colleagues addressed these concerns in the BMT-CTN 0901 trial, a comparison of MAC vs RIC (or non-ablative conditioning) in patients with AML and MDS with less than 5% myeloblasts, with the hypothesis that the reduced intensity conditioning might mitigate TRM while preserving anti-leukemic efficacy of alloHSCT (62). Younger patients (≤65 years of age) in complete remission (CR) prior to alloHSCT were randomized in a 1:1 fashion to MAC or RIC. Among AML patients, relapse rates were significantly higher in the RIC arm compared to the MAC arm (48.3% vs 13.5% respectively, P <.001), while TRM was significantly higher in MAC vs RIC (15.8% vs 4.4%, P = .002). Overall survival was not significantly different between the arms but was numerically improved in the MAC arm. The significant difference in relapse in favor of MAC led to the early termination of this randomized trial, and established MAC as the preferred conditioning strategy in younger, fit patients with AML. The BMT-CTN 0502 trial established RIC alloHSCT as a treatment option in older, frailer patients not suitable for MAC alloHSCT (63).

In an attempt to broadly augment the RIC regimen, the phase II FIGARO trial compared combination fludarabine/amsacrine/cytarabine-busulfan to fludarabine-based RIC regimen (64). Among 244 patients, 43% of whom were older than 60 years of age, no difference in OS was detected (hazard ratio [HR] 1.05, 85% confidence interval [CI], 0.80 to 1.38). Though MRD positivity by flow-cytometry was linked to increased risk of relapse, the authors did not identify an interaction between MRD, conditioning intensity, and survival. In contrast, an NGS-MRD analysis by Hourigan and colleagues of the BMT CTN 0901 trial suggested that MAC can overcome the poor survival associated with persistent residual molecular disease as compared to RIC (42). Importantly, the 0901 trial focused on largely younger patients who were eligible for MAC. Another multicenter retrospective analysis of pre-transplant NGS-MRD in older patients largely undergoing RIC transplantation suggested that MRD is prognostic, but that risk is largely encoded at the time of diagnosis, with MRD lacking independent prognostic value within diagnostic risk groups (25).

The critical question is whether there is a RIC regimen that can overcome the poor risk tied to diagnostic genetics and pre-transplant MRD. Alternative methods include augmenting RIC with the addition of selective small molecule inhibitors, such as venetoclax (“RIC-Ven”) in high-risk myeloid malignancies (65). In a phase I study done at our center adding 7 days of venetoclax to standard RIC Flu/Bu2 (fludarabine 30 mg/m2 QD and busulfan 0.8 mg/kg BID) on day -5 to day -2, followed by matched related or unrelated donor unmanipulated PBSC with tacrolimus/methotrexate GVHD prophylaxis, we observed that the addition of venetoclax to the conditioning was well tolerated, and had no deleterious effects on engraftment, infection, or GVHD. After a median of 14.7 month follow up (range, 8.6-24.8 months), progression-free survival (PFS) was 12.2 months (95% CI, 6.0-not estimable) and median overall survival was not reached among survivors. We performed dynamic NGS-MRD surveillance to track MRD conversion and to detect early relapse. Among the 18 patients with pre-transplant MRD, 9 of the 18 patients who experienced conversion to MRD negativity after transplant remain in complete remission. The true benefit of adding venetoclax to RIC alloHSCT will require a randomized study to assess the impact on relapse risk and survival as compared to traditional RIC regimens.

The prevention of GVHD in the form of immunosuppression peri- and post- alloHSCT has evolved from single-agent strategies to combination strategies using a calcineurin inhibitor (CNI) such as cyclosporine or tacrolimus, in conjunction with methotrexate, mycophenolate mofetil (MMF), and/or sirolimus (66–71). Most recently, the combination of post-transplant cyclophosphamide (PTCy), CNI, and MMF have led to improvement in GVHD-free, relapse-free survival in patients undergoing HLA-matched RIC alloHSCT as compared to tacrolimus and methotrexate in the randomized phase III BMT CTN 1703 trial (72). The results of this important study have already led to a shift in the GVHD prevention paradigm after RIC transplantation across the world. Although the reduction in GVHD, especially cGVHD, associated with PTCy does not appear to be associated with a higher rate of relapse in this and other studies, it remains unknown whether the GVL effect is truly preserved after PTCy, or whether the lower relapse rates are mediated by alternative mechanisms, such as NK cells, or other activity (73). More research is needed to understand the functional impact (if any) of post-transplant cyclophosphamide on T-cell mediated GVL.

## Immunology and limits of transplant and GVL

After the cytoreductive activity of conditioning chemotherapy and/or radiotherapy, the alloreactive donor immune system (largely T and potentially NK cells) mediates the anti-leukemic activity of alloHSCT which underpins the durable responses that are seen in a subset of AML patients. The presence of a GVL effect was first suggested by animal models, and then inferred from observations that patients with GVHD have reduced rates of, or delayed presentations of relapse as compared to patients who did not have GVHD (74–78). We refer the reader to the review from Sweeney and Vyas for a full discussion of the GVL effect, but note that additional lines of evidence for the presence of the GVL effect include the increased relapse rate after T-cell depleted transplantation, efficacy of RIC transplantation, efficacy of immunosuppression withdrawal and Donor Lymphocyte Infusions (DLI) as treatment for relapse, as well as loss of HLA antigens at relapse suggesting immune evasion mechanisms (61, 79–84).

## Targeted therapy in the post-transplant setting

### FLT3

The fms-related tyrosine kinase 3 gene (*FLT3*) encodes a receptor in the FMS family, and was originally implicated in leukemia biology in the early 1990s with northern blot analysis identifying high levels of expression in blasts (85). Several years later, internal tandem duplications (ITD) were identified in the *FLT3* gene in acute myeloid leukemia, with subsequent identification of activating D835 mutations within the activating loop of *FLT3* (86, 87). It is now estimated based on large patient datasets that *FLT3* mutations are identified in ~30% of newly diagnosed AML (8, 88). There are now several multi-kinase and FLT3 inhibitors approved for the treatment of *FLT3*-mutated AML in the pre-alloHSCT setting, and there has been interest in studying these inhibitors in the peri-and post-transplant setting, with relevant agents including sorafenib, midostaurin, gilteritinib, and quizartinib. Crenolanib, a potent inhibitor with promise against resistance mutations, is being studied in a single-arm, phase II study (NCT01468467) as maintenance therapy after allo-HSCT for up to two years (89).

### Sorafenib

Sorafenib is a multi-kinase inhibitor which was initially studied in leukemia cell lines as a potent inhibitor of FLT3 enzymatic and signaling activity (90). Chen and colleagues first showed the safety and tolerability of incorporating sorafenib therapy after alloHSCT in *FLT3*-ITD mutated AML in a phase I trial (91). In this study of 22 patients, sorafenib was initiated between days 45 and 120 after alloHSCT and continued in twelve 28-day cycles. One-year PFS was 85%. Pratz and colleagues next prospectively studied sorafenib in the peri-transplant period in 44 patients, 21 of whom started sorafenib pretransplant (92). Event free survival (EFS) at 2 years was 74%. Sorafenib was subsequently studied in phase II and III clinical trials, which confirmed its benefit. The SORMAIN phase II trial randomized 83 patients with *FLT3-*ITD+ AML undergoing alloHSCT in CR to receive sorafenib for 2 years versus placebo (93). The primary endpoint of relapse-free survival (RFS) was 53.3% with placebo compared to 85% with sorafenib, with a significant HR for relapse or death of 0.39 (95% CI, 0.18 to 0.85; log-rank *P* = .013) with median follow-up of 41.8 months. Of note, the median duration of therapy in the sorafenib group was 34.57 weeks, with side effects representing the most common reason for sorafenib discontinuation. Acute and chronic GVHD represented the most common grade 3+ adverse events, 76.8% in the sorafenib arm and 59.8% in the placebo arm. Other common grade 3+ adverse events in the sorafenib arm included infections in 26.2%, GI toxicity in 14.3%, and electrolyte derangements in 14.3% of patients. Sorafenib was studied in another randomized trial, a phase III study of 202 adult patients with *FLT3*-ITD+ AML who received alloHSCT and had hematopoietic recovery within day +60 who were randomized to sorafenib or non-maintenance control at 30-60 days post-alloHSCT (94). The original primary endpoint of 1-year cumulative incidence of relapse (CIR) in the intention-to-treat population was 7% in the sorafenib group and 24.5% in the control group (HR 0.25, 95% CI 0.11-0.57; p=0.0010). Long term follow-up showed significantly improved OS (72% sorafenib vs 55.9% placebo, HR 0.55, 95% CI 0.34-0.88; p=0·011) as well as improved leukemia-free survival (LFS), lower CIR, and no increase in NRM (95). These studies established 2-year sorafenib maintenance therapy as standard of care in *FLT3*-ITD mutated patients for long term disease control after transplant and is now recommended by the AML guideline of the National Comprehensive Cancer Network (NCCN) and ELN. As discussed below, sorafenib may act not only via cytoreduction, but via IL15-mediated enhancement of the GVL effect (96).

### Midostaurin

Midostaurin is a multi-kinase inhibitor that was initially studied in humans as monotherapy in those not felt to be candidates for intensive induction, and is now approved in combination with cytotoxic chemotherapy in the up-front treatment of AML (97, 98). The *post hoc* efficacy analysis of midostaurin-maintenance in the RATIFY trial similarly did not confirm relapse risk reduction though in a non-transplant setting, thereby limiting the FDA label to its use in induction and consolidation phases of therapy. A phase II study investigated the addition of midostaurin to intensive chemotherapy, followed by alloHSCT and 1 year of single-agent maintenance (99). This study suggested EFS benefit to midostaurin, including in older patients. A subsequent prospective study, the RADIUS trial, of post-alloHSCT midostaurin in patients with hematologic recovery received midostaurin in twelve 4-week cycles. The primary endpoint of RFS at 18 months post-alloHSCT was numerically increased: 89% in the midostaurin arm compared to 76% in the standard of care arm. However, neither RFS nor OS was improved in a statistically significant manner in this negative trial (100). The most common grade 3+ adverse events in the midostaurin arm included hypertension and AST/ALT increase each in 13% of patients, as well as nausea in 10% of patients. Rates of GVHD were similar in the midostaurin (70%) compared to the standard of care arm (73%).

### Gilteritinib

Gilteritinib is a selective FLT3 inhibitor which targets both ITD and tyrosine kinase domain (TKD) mutations, as well as exhibiting multikinase inhibitor activity against c-Kit and AXL, which has been implicated in resistance mechanisms to *FLT3* inhibitors (101, 102). Approval in the relapse/refractory setting was based on the ADMIRAL trial, and a retrospective study evaluated gilteritinib in this patient population post-alloHSCT (103, 104). A follow-up to the ADMIRAL trial reported that 18 of 26 long-term survivors without relapse in the gilteritinib arm proceeded to alloHSCT, with 16 of 18 re-starting gilteritinib in the post-alloHSCT setting (105). In a report focusing on post-alloHSCT outcomes in the ADMIRAL trial, patients who resumed gilteritinib and had a CR had low relapse rates (20% for those in CRc, 0% for those in CR) (106). The unpublished phase III MORPHO study (BMT CTN 1506) randomized 356 patients to two years of gilteritinib (120mg per day) starting 30-90 days post-alloHSCT vs placebo in adults with *FLT3-*ITD AML in CR1. In this negative trial, there was no statistically significant difference in the primary endpoint of RFS or OS. However, there was a suggestion that gilteritinib had RFS benefit in MRD-positive patients (HR=0.515, 95% CI: 0.316, 0.838, p = 0.0065), in contrast to patients without detectable MRD (HR=1.213, 95% CI: 0.616, 2.387, p = 0.575) based on a prespecified subgroup analysis. Key adverse events included neutropenia (42.1% vs 15.8% in placebo) and chronic GVHD (52.2% vs 42.1% in placebo). It is unclear why MORPHO did not show benefit in the overall population, in contrast to the two positive studies for sorafenib. Possibilities include differences in TKI (with sorafenib having more salutary off-target effects), differential impacts on GVL possibly through IL15, insufficient power/sample size, or gilteritinib levels influenced by triazole use across different centers.

### Quizartinib

Quizartinib has been studied in a phase II study as monotherapy in R/R *FLT3*-ITD+ AML and combined with ara-C in older patients with AML (107, 108). The QuANTUM-First randomized phase III trial evaluated quizartinib in patients with *FLT3*-ITD AML with or without alloHSCT, and a *post-hoc* analysis presented at EHA 2023 (Abstract S137) suggested benefit in those receiving alloHSCT (109). Sandmaier and colleagues conducted a phase I study of quizartinib maintenance after transplantation in 13 patients, reporting 1 relapse (110). Grade 1-2 QTc prolongation was seen in 54% of patients in this study, and none had grade 3 QTc prolongation or other cardiac toxicity.

### GVL effects of FLT3 inhibition

FLT3 is a receptor tyrosine kinase which has established roles for the development of hematopoietic stem cells and early myeloid progenitors (111). There has been long-standing interest in understanding how mutated *FLT3* alters leukocyte cell function and cytokine production with implications for relapse prediction (112, 113). Metzelder and colleagues concluded from retrospective data that sorafenib may synergize with the GVL effect based on differential rates of responses and outcome in relapse after alloHSCT as compared to relapses after chemotherapy (114). Tschan-Plessl and colleagues extended these observations by noting in their retrospective analysis that patients who developed chronic GVHD had a lower risk of developing sorafenib resistance (115). Immunophenotyping of three patients with *FLT3*-ITD mutated AML who relapsed after alloHSCT implicated a direct effect of sorafenib on immune function through CD8+PD1+ lymphocytes (116). Mathew and colleagues found that sorafenib exposure mediates reduced ATF4 expression leading to IRF7-IL15 axis activation, in turn leading to anti-AML lymphocytes with features suggesting longevity and metabolic reprogramming (96). Zhang and colleagues studied the more selective FLT3 inhibitor gilteritinib and its effects on lymphocytes, finding that gilteritinib exposure reduced co-inhibitory receptors on CD8+ T cells and up-regulated IL-15 expression (117). In total, there is growing data supporting the notion that FLT3 inhibition may have salutatory off-target effects on the donor graft which enhances the GVL effect, and in fact may become relevant as modulators of Chimeric Antigen Receptor (CAR) T cell therapies targeting *FLT3* mutated AML (118, 119). Whether these salutary off-target effects are similar, or the extent to which they exist, across the different available FLT3 targeted therapies remains to be elucidated.

### Unanswered questions and next steps for FLT3

A major unanswered question in this patient population is the utility of post-alloHSCT maintenance with FLT3 inhibitors in the setting of up-front FLT3 inhibitor use, particularly in the absence of detectable pre-transplant MRD. This is particularly salient as few patients (9 out of 83) in the SORMAIN trial were treated with up-front midostaurin as part of their induction regimen as is now standard of care. The MORPHO data strongly suggest that post-transplant gilteritinib maintenance provided benefit to the 50% of patients with detectable MRD either pre- or post-transplant compared to those without detectable MRD. Up-front inhibition of FLT3 may be enough to achieve MRD-negativity in a subset of patients. Subgroup analysis from SORMAIN and MORPHO differ in this regard. Exploratory analysis from SORMAIN suggested that patients with undetectable MRD before alloHSCT derive the most benefit from the multi-kinase TKI sorafenib. This is in contrast to the MORPHO study which suggested that only patients with MRD-positivity derived benefit from gilteritinib. Biologic rationales for both contradictory findings have been proposed. Patient and disease-related factors including prior treatment history (i.e., inclusion of FLT3 inhibitor with frontline induction chemotherapy; midostaurin approval was in 2017) and co-mutations contribute to the challenges in interpreting these studies. Ultimately, adequately powered prospective randomized trials in the relevant MRD subgroups will be required to address this important question. Further, while there is retrospective data and laboratory evidence for connections between FLT3 inhibition and the GVL effect, these findings are not clinically actionable at present. A key unanswered question is whether FLT3 inhibitors differ in any pro-GVL effect, and if so, what underlies that difference. One hypothesis includes the degree to which different FLT3-inhibitors exhibit multikinase activity, and activate the IRF7/IL-15 axis, but this needs to be more rigorously studied.

Lastly, while the RATIFY trial included patients with tyrosine kinase domain (TKD) *FLT3* mutations, all of the randomized studies of FLT3-inhibitor maintenance after alloHSCT have focused on *FLT3*-ITD mutated AML. Thus, it is unclear whether patients with *FLT3*-TKD benefit from TKI maintenance post-transplantation, although MRD-positive patients may represent a subgroup at high risk of relapse who may derive particular benefit.

Take-home message:

- Sorafenib has demonstrated RFS and OS benefit in the post-transplantation maintenance setting and is a standard of care option for *FLT3*-ITD mutated AML.
- Patients with detectable *FLT3* mutation at the pre-transplant stage benefit from pre-emptive post-alloHSCT gilteritinib therapy.

### IDH1/2

Isocitrate Dehydrogenase (IDH) is a key enzyme in the citric acid cycle. Mutations in the gene coding for cytosolic IDH (*IDH1)* were first implicated in AML biology in 2009 when massively parallel sequencing identified *IDH1* mutations associated with normal cytogenetic status (120). Subsequently, mutations in the mitochondrial IDH (*IDH2)* were identified in AML (121). The pathogenic mechanism in AML was revealed to be similar to that in gliomas, in which neomorphic activity leads to generation of the oncometabolite 2-hydroxyglutarate from alpha-ketoglutarate, which in turn inhibits alpha-ketoglutarate dependent dioxygenases which impacts histone demethylation and leads to impaired cell differentiation (122–126). Bill and colleagues have published data showing that persistence of *IDH1* and *IDH2* mutations at the time of transplantation is linked with increased risk of relapse, and that this association may be mutation-specific (43). However, these mutation-specific findings were based on a small cohort (33 patients with detectable *IDH* MRD) and must be interpreted with caution. Nonetheless, it is likely that the impact of NGS-MRD will depend on co-mutation patterns and possibly in a mutation-specific manner. This is illustrated by data presented by Gui and colleagues at the American Society of Hematology 2023 annual meeting (Abstract 424), showing that *IDH2*-MRD pre-transplant is prognostic for post-transplantation outcomes in the absence of co-mutated *FLT3* or *NPM1*, but not in *FLT3* or *NPM1* co-mutated cases.

The mutant IDH1 inhibitor ivosidenib has been studied in a multicenter phase I trial as maintenance after alloHSCT (127). Ivosidenib was initiated between days 30 and 90 in 16 patients and continued for up to twelve 28-day cycles. In this study, the 2-year CIR was 19% and 2-year PFS and OS were 81% and 88%, respectively. Similarly, the mutant IDH2 inhibitor enasidenib was also studied as post-alloHSCT maintenance in a multi-center phase I trial in which 19 patients with myeloid malignancies received up to twelve 28-day cycles of treatment (128). Twelve-month CIR was 16% with 2-year PFS and OS of 69% and 74% respectively. Grade 3+ toxicities were rare in these trials, without any apparent increase in GVHD or infections. There is an ongoing phase I trial focusing specifically on enasidenib prophylactic maintenance in patients with *IDH2*-mutated AML (NCT03728335).

### Unanswered questions and next steps for IDH1/2

An advantage of the IDH1 and IDH2 inhibitors in clinical use is their selectivity for the mutant enzyme, a property likely required for their use in humans given the central role these enzymes play in energy metabolism across tissues. Little is known about any off-target effects on the immune system or the GVL effect. However, data from Notarangelo and colleagues suggests that the D-2HG oncometabolite negatively impacts cytotoxic T cell activity via dysregulation of glucose metabolism and decreased IFN-gamma (129). An intriguing possibility is that post-transplant or peri-CAR-T inhibition of mutant IDH may ameliorate an inhibitory microenvironment mediated by 2-HG, allowing for maximal GVL effect. Other data suggests that *IDH*-mutated AML may be particularly susceptible to NK-based cellular therapies due to down-regulation of inhibitory HLA Class I proteins (130). These preclinical data highlight the importance of ongoing basic immunologic research to delineate the potentially differential impact IDH inhibitors may have on various arms of the immune system. This is of particular importance as combination therapy with hypomethylating agents may modulate the transcriptional and immunomodulatory effects of oncometabolite concentrations. Multiple resistance pathways may be involved in *IDH*-mutated AML, lending rationale to testing combination strategies (131).

Take-home message:

- Prophylactic IDH1/2 inhibitor maintenance therapy in the post-transplant setting has not yet been established, but the safety data and preliminary efficacy suggest that this is feasible.
- With the addition of venetoclax and IDH1/2 inhibitors to frontline older AML therapies, the role of maintenance therapy remains in question and consideration of co-mutations or potential for resistance might be necessary.

### DNMT inhibitors

Parenteral DNA methyltransferase inhibitors (DNMTi) or hypomethylating agents (HMA) (azacitidine, decitabine) and more recently oral hypomethylating agents (azacitidine/CC-486, and cedazuridine/decitabine) have been extensively studied in the treatment of myeloid malignancies. In the transplant setting, hypomethylating agents have been studied in combination with traditional chemotherapy during conditioning, as maintenance post-alloHSCT, and in the post-alloHSCT relapse setting.

*Conditioning*: there are few randomized trials evaluating the efficacy of adding DNMTi to conditioning regimens. Xuan et al. report on a phase III trial of G-CSF, decitabine and busulfan/cyclophosphamide vs busulfan-cyclophosphamide in patients with MDS or secondary AML (132). The primary endpoint of this study was 2-year CIR, which was 10.9% in the G-CSF, decitabine, and busulfan-cyclophosphamide (Bu-Cy) arm compared to 24.8% in the control arm, with a statistically significant HR of 0.39 (95% CI 0·19-0·79; p=0·011). This finding builds on earlier phase studies adding decitabine to MAC Bu-Cy conditioning that demonstrated benefits in AML patients, even those transplanted with active disease (133, 134). In the non-randomized setting, there are studies which report on the addition of azacitidine to RIC for high risk MDS and for older patients with AML (CALGB 100801) (135), addition of 5-day decitabine to conditioning in patients with MDS and MPN (136), decitabine induction with MAC in high-risk patients (with infectious complications highlighted in this study) (137), and the use of 10-day decitabine with fludarabine and TBI (138).

*Maintenance:* Hypomethylating agents have been given preemptively after alloHSCT in cases of decreasing chimerism or MRD positivity or as prophylactic maintenance. Platzbecker and colleagues reported results of the RELAZA trial, in which patients were treated with azacitidine when donor CD34+ chimerism fell below 80% in patients with MDS and AML (139). In the RELAZA trial, 13 out of 20 patients (65%) still relapsed at a median of 231 days despite azacitidine being pre-emptively administered for dropping chimerism. A subsequent phase III trial randomized 187 patients to 32 mg/m^2^ of azacitidine given for 5 days every 28 days (up to 12 cycles) to no treatment in high-risk AML and MDS (140). A median of 4 treatment cycles of azacitidine were administered in the interventional arm, with approximately a quarter of patients finishing all planned cycles of azacitidine. The most common reasons for azacitidine maintenance discontinuation were relapse (47% of discontinuations), followed by toxicity (18%) and patient preference (15%). This trial was negative, with no statistically significant difference in RFS or OS. The toxicities in this trial are emblematic of the delicate balance between efficacy and tolerability in post-transplant maintenance, as 19.2% of patients experienced grade 3+ hematologic AE, compared with 2.3% grade 3+ hematologic AE in the control group. There were no major differences in GVHD between the azacitidine and control groups, with D100 cumulative incidence of grade 2-4 aGVHD of 25.5% with azacitidine and 28.7% in the control group (P = .73) and no difference in 1-year rates of chronic GVHD (25.8% in the azacitidine maintenance arm compared to 30.8% in the no maintenance arm). Oral formulations of DNMTi (i.e. oral azacitidine (CC-486) or oral decitabine/cedazuridine) result in lower peak plasma concentrations hence lower cumulative daily exposure, which may result in sustained epigenetic regulation compared to subcutaneous or intravenous formulations. Trials with these oral formulations for pre-emptive or prophylactic post-transplant maintenance are ongoing (refer to **Table 1**).

**Table 1:** Non-randomized trials of augmented conditioning and post-transplantation maintenance.

Trial ID (NCT or other)/Reference	Agent/Dose/Schedule	Strategy	Study Type	Patient Population (Key Inclusion/Exclusion)	N	Primary Outcome	Key Secondary Outcomes	Key Toxicities
***FLT3***								
NCT01398501; Chen et al. BBMT, 2014 (PMID: 25239228) (91)	Sorafenib. S*tart:* between D+45 & D+120 (median D+69.5). *Dose*: 200-400mg BID. *Duration:* Up to 12 28-day cycles. *Completed all planned cycles:* 9/22 (40.9%).	Prophylactic Maintenance	Phase I	Adults age 18 - 75, ECOG 0-2 with *FLT3*-ITD+ AML undergoing first allo-HSCT with 7/8 or 8/8 donors who are in morphologic CR after day +30. Donor chimerism ≥ 70% (peripheral blood) and adequate blood counts (ANC ≥1000, PLT ≥ 50,000) are required. Active GVHD requiring prednisone ≥ 0.5mg/kg/day is exclusionary.	22	MTD: 400mg BID	PFS (1-year): 85% OS (1-year): 95%	Anemia: 5/22 (22.7%), thrombocytopenia: 2/22 (9%), leukopenia: 2/22 (9%). 12-mo CI cGVHD from starting sorafenib: 38%
NCT01477606; Schlenk et al. Blood, 2019 (PMID: 30563875) (99)	Midostaurin. *Start:* between D+30 & D+100 (median D+71). *Dose:* 50mg BID. *Duration:* 1 year. *Completed all planned cycles:* 41.3%, median time on maintenance: 9 months.	Prophylactic Maintenance	Phase II	Adults age 18 - 70 with *FLT3*-ITD+ non-APL AML. CBF-alterations excluded.	75 (after allo-HSCT)	EFS: Improved (as well as OS) in landmark analysis.	2 year CI-Relapse: 13.3%	"Blood/Marrow" toxicity: 52%. Infection: 56%. Febrile Neutropenia: 13%
NCT01468467; Sandmaier et al. Am J Hematology, 2018 (PMID: 29090473) (110)	Quizartinib. *Start:* between D+30 and D+60. *Dose:* 40mg and 60mg/day in 3+3 design. *Duration:* 28 day cycles x24 (~2 years). *Completed all cycles:* 38%	Prophylactic Maintenance	Phase I	Adults age ≥ 18 with *FLT3*-ITD+ AML in CR1 or CR2 after HLA-matched allo-HSCT with donor CD3 chimerism ≥ 50% and adequate blood counts: ANC > 1000, PLT > 50,000. Recent active CNS leukemic involvement or active grade ≥2 GVHD were exclusionary.	13	MTD: None identified. 60mg/day selected	1 relapse reported	Anemia: 15%. Neutropenia: 23%. Thrombocytopenia: 15%. GVHD: 8%
NCT02400255; Unpublished	Crenolanib. *Start*: between D+42 and D+90. *Dose:* 100mg TID. *Duration:* 2 years	Prophylactic Maintenance	Phase II	Adults age ≥18 with *FLT3*-ITD or *FLT3*-D835-mutated AML. First allo-HSCT with no more than 1 mismatched donor. In CR post-allo-HSCT with adequate blood counts. aGVHD ≤ grade 1, no more than mild/limited cGHVD.	NA	2-year relapse rate	N/A	Not yet available
***IDH1/2***								
NCT03564821; Fathi et al. Clin Cancer Res, 2023 (PMID: 37014667) (127)	Ivosidenib. *Start*: between D+30 and D+90. *Dose:* 500mg qD. *Duration:* 12 28-day cycles (~1 year)	Prophylactic Maintenance	Phase I	Adults age 18 - 75 with *IDH1* R132-mutated AML, CMML, or MDS with normal organ and marrow function in CR post allo-HSCT. Patients with QTC > 500 or risk of QTC prolongation were excluded.	18	RP2D: 500mg qD	2-year CI Relapse: 19%. 2-year CI NRM: 0%. 2-year OS: 88%. 2-year PFS: 81%	6-month CI of grade II-IV aGVHD: 6.3%. 2-year CI of all cGVHD: 63%
NCT03515512; Fathi et al. Blood Advances, 2022 (PMID: 36150050) (128)	Enasidenib. *Start*: between D+30 and D+90. *Dose*: starting dose 50mg qD. *Duration*: 12 28-day cycles (~1 year)	Prophylactic Maintenance	Phase I	Adults age ≥18 with *IDH2*-mutated AML, CMML, or MDS. If AML, must be in CR or CRi. Must have normal organ and marrow function (ANC >1000, PLT > 50,000).	23	RP2D: 100mg qD	2-year Relapse: 16%. 2-year PFS: 69%. 2-year NRM: 16%. 2-year OS: 74%	Treatment related AE. Grade 3+ Anemia: 8.7%, Grade 3+ neutropenia 4.3%. . 6-month g2 aGVHD: 16%. 1-year cGVHD: 68%. 1-year moderate/severe cGVHD: 42%
NCT03728335; Unpublished	Enasidenib. *Duration*: 24 28-day cycles (~2 years)	Prophylactic Maintenance	Phase I	Adults age ≥18 with *IDH2*-mutated AML and adequate end organ and marrow function.	15 (actual enrollment)	Incidence of AE	OS, LFS, Relapse, NRM, GRFS	Not yet available
NCT04522895; Unpublished	Enasidenib. *Start*: between D+30 and D+65. *Dose*: 100mg qD. *Duration*: 12 28-day cycles (~1 year)	Prophylactic Maintenance	Phase II	Adults age ≥18 with *IDH2*-mutated MDS, CMML and AML in remission (CR) after allo-HSCT with hematologic recovery post-alloHSCT. No active, steroid-refractory GVHD.	50 (estimated enrollment)	Incidence of AEs	OS, RFS, NRM, Relapse, GVHD	Not yet available
***DNMTi ± Small Molecule***								
CALGB 100801; Vij et al. BBMT, 2019 (PMID: 31212080) (135)	Azacitidine. *Start*: between D+42 and D+90 (median D+61). *Dose*: 32mg/m2 SC × 5 days. *Duration*: up to six monthly cycles. *Completed all planned cycles*: 41%	Prophylactic Maintenance	Phase II	1) AML age 60 - 75 in MLFS who went to matched allo-HSCT within 6 months of documented CR. 2) MDS age <75 years with high risk features and <10% BM blasts.	63 (evaluable)	2-year PFS: 41.2%	2-year NRM: 33.4%. 2-year CI Relapse: 25%. 2-year OS: 45.7%. Reasons for discontinuation: Refusal (33%), Progression (25%), AE (21%), Death (17%)	Grade 3-4 Hematologic AE: 95.2%. Grade 3-4 Non-Hematologic AE: 69.8%. Grade 5 Non-Hematologic AE: 27%. 100 day CI grade III-IV aGVHD: 12.7%. 2-year rate of any cGVHD: 30.2%, extensive cGHVD: 14%
NCT00422890; RELAZA: Platzbecker et al. Leukemia, 2012 (PMID: 21886171) (139)	Azacitidine. *Start*: upon decrease in chimerism <80%, median 13 days from MRD+. *Dose*: 75 mg/m2 daily for 7 days every 28-30 day cycle. *Duration*: 4 cycles	Pre-emptive Therapy	Phase II	Adults age ≥ 18 years with CD34+ MDS or AML undergoing allo-HSCT. If donor CD34+ chimerism < 80%, offered azacitidine. Morphologic relapse (i.e. BM blasts ≥ 5%) around the time of chimerism drop and organ impairment (hepatic, renal) were excluded.	20 MRD+/ 59 screened	Major response after completing four cycles of azacitidine (i.e. rise of chimerism ≥80%): 50% (10/20)	Minor response (no increase in chimerism, but without relapse): 30%. Rate of subsequent hematologic relapse after chimerism decrease: 65%	Grade 3-4 Neutropenia: 80%. Neutropenic Fever: 20%. Grade 3-4 Thrombocytopenia: 65%. Dose reduction for myelosuppression: 45%. GVHD in those without prior history of GVHD: 0%
NCT05788679; Unpublished	Azacitidine	Pre-emptive Therapy	Phase II	Adults age ≥ 18 with MDS, MDS/AML or AML w/ MDS-related changes with 20-29% blasts undergoing allo-HSCT. Those without a genetic MRD marker were excluded.	200 (estimated enrollment)	Relapse or death within 1 year from first MRD+ sample	Rate of MRD negativity. Severity of GVHD. RFS. OS	Not yet available
#ISRCTN36825171; RICAZA: Craddock et al, BBMT, 2016 (PMID: 26363443) (141)	Azacitidine. *Start*: earliest D+42, median D+54. *Dose*: 36mg/m2 SQ on D1-5 of every 28-day cycle. *Duration*: up to 12 months after transplantation. *Completed all planned cycles*: 46% (17/37).	Prophylactic Maintenance	Phase I	Adult patients with AML undergoing RIC allo-HSCT. To initiate Azacitidine, required stable engraftment with ANC > 1000 and PLT > 50,000.	51	Safety and tolerability of AZA	1-year OS: 81%. 1-year RFS: 57%. 1-year NRM: 8%	Grade 3-4 anemia: 10%. Grade 3-4 thrombocytopenia: 13%. Grade 3-4 neutropenia: 10%. Any aGVHD: 33% (no grade 3-4). Any cGVHD: 19.6% (none extensive).
NCT01835587; de Lima et al. BBMT, 2018 (PMID: 29933073) (142)	Azacitidine (oral CC-486). *Start*: D+42 to D+84. Dose: cohort 1: 200mg daily for 7 days per 28-day cycle. Cohort 2: 300mg daily for 7 days per 28-day cycle. Cohort 3: 150mg daily for 14 days per 28-day cycle. Cohort 4: 200mg daily for 14 days per 28-day cycle. *Duration*: 12 cycles, median 9 cycles across the entire cohort. *Completed all planned cycles*: 43%.	Prophylactic Maintenance	Phase I/II	Adults age ≥ 18 years with MDS or AML undergoing allo-HSCT in morphologic CR with ANC > 1000 and PLT > 75,000 prior to CC-486 initiation. Exclusion: grade ≥2 GVHD, GI GVHD, other active malignancies, use of hypomethylating agents or lenalidomide/pomalidomide/thalidomide post-allo-HSCT.	30 (ITT population)	MTD: not reached. Cohort 4 expanded	1-year relapse rate: 21%. 1-year OS in 7 day cohort: 86%. 1-year OS in 14 day cohort: 81%.	Grade 3-4 anemia: 13% overall, 16% in cohort 4. Grade 3-4 thrombocytopenia: 10% overall, 11% in cohort 4. Grade 3-4 neutropenia: 17% overall, 21% in cohort 4. 1-year CI aGVHD or cGVHD: 50%. Chronic GVHD: 30%
NCT05823714; Unpublished	Azacitidine+Venetoclax+ modified BuCy. *Dose*: Venetoclax: 200mg/day for 7days. Azacitidine: 75mg/ m²/day for 7days	Augmented Conditioning	Phase II	Individuals age 8 - 65 with high risk MDS or AML without uncontrolled infection.	70 (estimated enrollment)	DFS, OS	Not yet available: VOD, GVHD, TRM	Not yet available
NCT03613532; Garcia et al. Blood Advances, 2024 (PMID: 38197938) (143)	RIC-Venetoclax + Azacitidine/Venetoclax maintenance. *Start*: between D+42 and D+90. *Dose*: azacitidine 36mg/m2 IV on D1-D5 of each cycle. Venetoclax: 400mg PO qD on D1-14 of each cycle. *Duration*: 1 year (8 42-day cycles or 12 28-day cycles). *Median number of cycles:* 42-day cycle cohort = 3 of 8, 28-day cycle cohort = 5.5 of 12. *Completed all planned cycles*: 40.9%	Prophylactic Maintenance	Phase I	Adults age ≥ 18 with high risk MDS, MDS/MPN, or AML not eligible for MAC allo-HSCT. Eligible to initiate maintenance if patients had no morphologic disease (BM blasts < 5%) at screening, adequate engraftment/count recovery; ANC > 1000, PLT >50,000, and no grade 2-4 GVHD on prednisone ≥ 0.5 mg/kg daily.	27	MTD: none. RP2D (28-day cycle): azacitidine 36 mg/m2 D1-5, venetoclax 400 mg qD D1-14. Safety, Tolerability	2-year OS: 58%. 2-year PFS: 52%. 2-year CI relapse: 48%. NRM: 0%	Grade 3-4 anemia: 45.5%. Grade 3-4 thrombocytopenia: 77.3%. Grade 3-4 leukopenia: 95.5%, neutropenia: 81.8%. CI of grade II to IV acute GVHD at 6 months: 22% 1-year CI of chronic GVHD: 23%
NCT03613532; Unpublished	RIC-Venetoclax + oral decitabine/cedazuridine/venetoclax maintenance	Prophylactic Maintenance	Phase I	Adults age ≥ 18 with high risk MDS, MDS/MPN, or AML not eligible for MAC allo-HSCT. Eligible to initiate maintenance if patients had no morphologic disease (BM blasts < 5%) at screening, adequate engraftment/count recovery; ANC > 1000, PLT >50,000, and no grade 2-4 GVHD on prednisone ≥ 0.5 mg/kg daily.	N/A	MTD	Not yet available: OS, PFS, ORR, relapse rate, NRM, GVHD	Not yet available
NCT04809181; Unpublished	Azacitidine + Venetoclax	Pre-emptive Therapy	Phase II	Adults with AML or MDS who are MRD-positive (by MFC, fusion gene PCR, or gene mutation) after allo-HSCT. Patients previously known to be resistant to azacitidine or decitabine or venetoclax are excluded.	95 (estimated enrollment)	RFS	OS, GVHD	Not yet available
NCT01700673; Webster et al. Leuk Lymphoma, 2021 (PMID: 34284701) (144)	Azacitidine & GM-CSF: *Start*: Between D+87 to D+215 (median D+133). *Dose*: Aza 37.5 - 75 mg/m2, GM-CSF D1-10 of each cycle. *Duration*: 12 cycles (median cycles received = 8). *Completed all planned cycles*: 27.8%	Prophylactic Maintenance	Phase II	Patients age ≥ 6 months with poor risk AML or MDS in CR post-allo-HSCT with adequate blood counts (ANC > 1000, PLT > 50,000) and without grade 3-4 aGVHD.	18 (received ≥1 dose)	2-year RFS: 47%. Median RFS: 18.6 months	Median OS: 29 months. 2-year OS: 57%	Grade 3-4 anemia: 11%. Grade 3-4 thrombocytopenia: 5%. Grade 3-4 neutropenia: 11%. Grade 3-4 GVHD: 5%
NCT05270200; Unpublished	Azacitidine + Chidamide. *Dose*: Azacitidine: 100mg D1-D5 of 28-day cycle, up to 6 cycles. Chidamide: 5mg for up to 2 years	Prophylactic Maintenance	Phase I/II	Patients age 18 - 60 with poor risk AML with post-transplant engraftment (ANC > 1500, PLT > 80,000) and without uncontrolled infection or grade 3-4 GVHD.	20 (estimated enrollment)	Safety, 1-year CI relapse	RFS, OS, GVHD	Not yet available
NCT02124174; Unpublished	Azacitidine + Valproic Acid. *Dose*: Azacitidine: 40mg/m2 daily on D1-D5. Valproic acid: 15mg/kg daily on D6-D28. *Duration*: 4 cycles (28-day cycles)	Prophylactic Maintenance	Phase II	Patients age 2 - 89 years of age with relapse/refractory AML or high risk MDS undergoing allo-HSCT 40-60 days prior to starting treatment with adequate organ function and blood counts (ANC > 1500 and PLT > 50,000).	50 (estimated enrollment)	1-year survival	Relapse rate	Not yet available
N/A; Guillaume et al. BMT, 2019 (PMID: 31089280) (145)	Azacitidine + DLI. *Duration*: median 5 cycles. *Completed all planned cycles*: 33%	Prophylactic Maintenance	Phase II	Patients with high-risk AML or MDS.	30	2-year OS and DFS: 65.5%	2-year CI of relapse: 27.6%	2-year CI of grade 1-3 aGVHD: 31.5%. 2-year CI of cGVHD CI: 53%
NCT03793517; Unpublished	Decitabine+modified BuCy. *Dose*: decitabine 200mg/m2 on days -12 and -11 pre-HSCT	Augmented Conditioning	Phase II/III	Patients age 18 to 55 with MLL-r, TLS-ERG, or SIL-TAL1 acute leukemia, with detectable MRD pre-allo-HSCT.	55 (estimated enrollment)	1-year CI Relapse. 2-year CI Relapse	1-year OS, NRM, OS, LFS, GVHD	Not yet available
NCT01277484; Han et al. J Hematol Oncol, 2015 (PMID: 26497198) (146)	Decitabine. *Start*: between D+42 and D+90 after allo-HSCT. *Dose*: 5 -15 mg/m2/day, D1-5, cycle = 4-week. Adaptive design, started 6-10 weeks after allo-HSCT.	Prophylactic Maintenance	Phase I	Patients with high risk MDS or MDS/AML in CR post-alloHSCT within 2 weeks of treatment and acceptable organ function with PLT ≥ 30,000 and ANC ≥ 1000. Patients with uncontrolled GVHD excluded.	15	"Dose and schedule finding of post-BMT Decitabine Treatment." 5mg/m2/day considered "most appropriate starting dose"	N/A	N/A
NCT00986804; Pusic et al. BBMT 2015 (PMID: 26055299) (147)	Decitabine. *Start*: enrolled between D+50 and D+100. *Dose*: escalating doses, 5mg/m2/d - 15mg/m2/d. *Duration*: D1-5 every 6 weeks, up to 8 cycles. *Completed all planned cycles*: 41% (9/22 evaluable)	Prophylactic Maintenance	Phase I	Adults age ≥ 18 years with AML or MDS in CR after allo-HSCT with adequate blood counts (ANC) ≥1,500, platelet count ≥50,000), and no grade 3-4 GVHD.	22 (evaluable)	MTD: none reached "dose of 10 mg/m2 for 5 days every 6 weeks appeared to be the optimal dose"	2-year OS: 56%. 2-year DFS: 48%. 2-year CI of relapse: 28%	Grade 3-4 anemia: 13.6% (3/22). Grade 3-4 thrombocytopenia: 59% (13/22). Grade 3-4 Neutropenia: 50% (11/22). Grade 3-4 GI GVHD: 9% (2/22).
NCT02252107; Cruijsen et al. BMT, 2021 (PMID: 33824442) (138)	Decitabine. *Dose/Schedule*: 20 mg/m2/day x10 days (From D-11 to D-2)	Augmented Conditioning	Phase II	Adults with poor or very-poor risk AML in CR1 after 7+3, eligible to receive allo-HSCT with non-myeloablative conditioning, without active infection, and who had not received prior hypomethylating agents.	46	1-year CI Relapse: 23%	1-year CI NRM: 11%. 1-year OS: 70%	Febrile neutropenia: 9%. Grade 3-4 aGVHD: 13%. Severe cGVHD: 20%
NCT05796570; Unpublished: MORE trial	Decitabine + Filgrastim. *Start*: between D+40 and D+120. Decitabine on D2 -D6 of 28-day cycle. Filgrastim on D1 - D6 of 28-day cycle	Prophylactic Maintenance	Phase II	Patients age 1 - 39 with AML, MDS, or therapy related myeloid neoplasm undergoing allo-HSCT with adequate organ function. Patients with *FLT3*-ITD excluded.	37 (estimated enrollment)	Feasibility Failure Rate	EFS, OS, Treatment Tolerability Rate	Not yet available
NCT06129734; Unpublished	Decitabine + Venetoclax. *Start*: must enroll by D+40. *Dose*: decitabine 5mg/m2 + venetoclax 400mg weekly. *Duration*: 1 year	Prophylactic Maintenance	Phase I/II	Adults age ≥18 with high risk MDS, MDS/AML, or AML in morphologic CR pre-allo-HSCT with adequate organ function. Prior progression on HMA/Ven or venetoclax therapy is exclusionary.	20 (estimated enrollment)	Safety, Feasibility	RFS	Not yet available
N/A; Zhang et al. Cancer Med, 2021 (PMID: 33932107) (148)	DLI ± Decitabine. *Start*: Prophylactic G‐CSF‐primed DLI between D+30 and D+90. *Dose*: decitabine 10mg/m2/day D1-5 with DLI on D7	Prophylactic Maintenance	Prospective, Single-Arm	Diagnosis of hematologic malignancy with unfavorable gene mutation receiving first allo-HSCT.	28 (with prophylactic DLI)	3-year CI post-DLI Relapse: 26.1%. CI aGVHD: 11% (grade 3-4 at 100 days). 3-year CI cGVHD: 21.6%	3-year NRM: 25%. 3-year OS: 48.2%. 3-year RFS: 48.9%	6-month CI of NRM: 17.9%. 1-year CI of NRM: 25%
ECT2012-003344-74; Kalin et al. Blood Advances, 2020 (PMID: 32936907) (149)	Decitabine + Panobinostat prior to DLI. *Dose/Schedule*: panobinostat monotherapy (20 mg) alone at days 1, 4, 8, and 11 of a 4-week cycle or in combination with. Decitabine (PNB/DAC20) at a dose of 20 or 10 mg/m2 on days 1 to 3 of every 4-week cycle. *Start*: D+28 post-allo-HSCT, intercalated with DLI	Prophylactic Maintenance	Phase I/II	Adults age 18 - 70 years with AML or high risk MDS with <10% bone marrow blasts at the time of allo-HSCT.	110	Feasibility	CI of relapse: 35%. 2-year OS: 50%. 2-year PFS: 49%	Grade 3/4 Bone Marrow AE: 13% at DAC20 dosing
NCT03454984; Unpublished	Guadecitabine (SGI-100) + DLI. *Dose*: 30/m2/day SQ for 5 days of 28-day cycle. guadecitabine to start between D+40 to D+130. *Duration*: 10 cycles	Prophylactic Maintenance	Phase II	Patients age 18 to 70 with MDS or AML with unfavorable genetics with <20% marrow blasts.	40 (estimated enrollment)	DFS	OS	Not yet available
***BH3 Mimetic/Apoptotic Pathway***								
NCT03613532; Garcia et al. Blood Advances, 2021 (PMID: 34614506) (65)	Venetoclax. *Dose*: 200-400mg from D-8 to D-3	Augmented Conditioning	Phase I	Adults age ≥ 18 with high risk MDS, MDS/MPN, or AML not eligible for MAC allo-HSCT who had a matched 8/8 donor. Prior venetoclax exposure allowed. In AML, must be in CR/CRi at study entry.	22	MTD: not reached. RP2D: 400 mg qD on D-8 through D−2. Safety	Median OS: not reached. Median PFS: 12.2 months. 1 year CI-Relapse: 37%. 1 year CI- NRM: 9.4%	Grade 3/4 Neutropenia: 27%. Febrile Neutropenia: 9%. Grade 3/4 Thrombocytopenia: 41%. 6 month CI grade 2-4 aGVHD: 23%. 1 year CI moderate/severe cGVHD: 27%
NCT05005299; Unpublished: VICTORY trial	Venetoclax + FluCy (NMA). *Dose/Schedule*: venetoclax: short-course on day -11 to -6. Venetoclax dose levels: Level A: 100mg qD administered on D-11 to D-6. Level B: 100mg qD on D-11, followed by 200mg qD on D-10 to -6. Level C: 100mg qD on D-11, followed by 200mg qD on D-10, 400mg qD on D-9 and 600mg qD administered on D-8 to D-6.	Augmented Conditioning	Phase I	Adults age 18 to 75 planning to undergo matched PBSC allo-HSCT for acute leukemia, MDS, CLL, NHL, plasma cell lymphoma with non-MAC.	18 (estimated enrollment)	DLT	GVHD, GRFS, Relapse, NRM, Chimerism	Not yet available
NCT05583175; Unpublished	Venetoclax + FluBu (RIC). *Dose*: venetoclax 100mg/d on D-10, 200mg on D-9 (first use and NR or untreated MDS), 400mg/ on D-8 - D+2 (7d).	Augmented Conditioning	Phase II	Patients age ≥55 years with high risk myeloid malignancies undergoing allo-HSCT with adequate organ function and without prior venetoclax ineffectiveness.	50 (estimated enrollment)	1-year and 2-year PFS	GVHD, relapse rate, OS, GRFS, NRM, EBV/CMV reactivation	Not yet available
NCT05807932; Unpublished	Venetoclax + FLAMSA + Treosulfan. *Schedule*: venetoclax D-11 to D-6 before stem cell infusion	Augmented Conditioning	Phase I/II	Adults age ≥ 18 with secondary AML, MDS, CMML with elevated blasts or high risk features undergoing first allo-HSCT. Patients with sAML with *FLT3*-mutation, or marrow blasts ≥30% excluded.	38 (estimated enrollment)	Safety	Graft failure, GVHD, VOD, EFS, Relapse, OS	Not yet available
***TP53***								
NCT03931291; Mishra et al. JCO, 2022 (PMID: 35816664) (150)	Eprenetapopt + Azacitidine. *Start*: between D+30 and D+100. Dose: eprenetapopt 3.7 g IV qD on D1-4 of each 28-day cycle. Azacitidine: 36 mg/m2 IV or SQ on D1-5 of each 28-day cycle. *Duration*: maximum 12 cycles, median received = 7 cycles. *Completed all planned cycles*: 39%	Prophylactic Maintenance	Phase II	Adults age ≥ 18 with *TP53*-mutated AML or MDS who were suitable candidates for first allo-HSCT and were in morphologic CR/CRi/CRp prior to maintenance initiation with engraftment (ANC ≥ 500, PLT > 20,000).	33 received maintenance (of 55 transplanted patients)	median RFS: 12.5 months. 1-year RFS: 59.9%	Median OS: 20.6 month. 1-year OS: 78.8%	Grade ≥3 anemia: 27%. Grade ≥3 thrombocytopenia: 36%. Grade ≥3 neutropenia: 27%. Any grade aGVHD: 12%. Any grade cGVHD: 33%
***Epigenetic Modifier***								
NCT01451268; PANOBEST: Bug et al. Leukemia, 2017 (PMID: 28751769) (151)	Panobinostat. *Start*: day ≥ +60. Dose: 10-20mg thrice weekly (Arm A), or every other week (Arm B). *Duration*: up to 1 year. *Completed all cycles as planned*: 52%	Prophylactic Maintenance	Phase I/II	Patients with high risk MDS or AML in CR post allo-HSCT.	42	MTD and RP2D: 20mg thrice weekly (Arm A) and 30mg thrice weekly (Arm B)	2-year CI relapse post 1st panabinostat dose: 20%. 2-year CI NRM post 1st panabinostat dose: 5%. 2-year OS: 81%. 2-year RFS: 75%	Related to maintenance treatment. Grade ≥3 anemia: 10% (Arm A), 0% (Arm B). Grade ≥3 thrombocytopenia: 29% (Arm A), 19% (Arm B). Grade ≥3 neutropenia: 10% (Arm A), 19% (Arm B). aGVHD rate: 9.5% (all in schedule A) 2-year CI of moderate/severe cGVHD: 29%
NCT05682755; Unpublished	Chidamide. *Dose*: range: 5mg - 20mg twice weekly. *Duration*: "terminal time 180 days after allo-HSCT"	Prophylactic Maintenance	Phase I/II	Adults age 18 - 65 with AML undergoing allo-HSCT in CR with adequate engraftment (ANC > 500, PLT > 20,000, Hgb > 8).	77 (estimated enrollment)	2-year PFS	100-day AE, NRM, OS, Relapse, GVHD	Not yet available
NCT03843528; Unpublished	Vorinostat + low-dose Azacitidine. *Dose/Schedule*: azacitidine 2 cycles of 32mg/m2/dose IV/SQ for 5 days, in 28 day cycles. Vorinostat: if azacitidine tolerated, added at a starting dose of 100mg/m2 on days 1-7 and 15-21 of each 28 day cycle (azacitidine continued). *Duration*: 7 cycles (9 total cycles of azacitidine)	Prophylactic Maintenance	Phase I	"Children and adolescents ages 1 to 21" with myeloid malignancies (AML, MDS, JMML, MPAL) undergoing allo-HSCT.	15 (estimated enrollment)	MTD	DLT, GVHD, Relapse, Survival, Immune Recovery	Not yet available
***XPO1***								
NCT02485535; Cooperrider et al. BMT, 2020 (PMID: 32376970) (152)	Selinexor. *Start*: beginning D+60 to D+100. *Dose*: 60 - 80mg on day 1 of each week or on days 1 and 3 of weeks 1-3. *Duration*: up to 12 28-day cycles. Median duration 224 days. *Completed all planned cycles*: 33% (4/12)	Prophylactic Maintenance	Phase I	Patients with AML and MDS who underwent allo-HSCT without grade 2-4 GVHD.	12	MTD and RP2D: 60 mg weekly	Median PFS: 775 days. 1-year PFS: 66%. Median OS: 872 days	Any grade aGVHD: 58%
***JAK/STAT pathway***								
NCT03286530; Unpublished	Ruxolitinib. *Dose*: BID. *Duration*: up to 24 28-day cycles	Prophylactic Maintenance	Phase II	Adults age 60 - 80 years with AML in CR receiving first RIC matched allo-HSCT.	64 (estimated enrollment)	1-year GRFS	PFS, OS, Relapse, TRM	Not yet available
***DRD2***								
NCT03932643; Unpublished	ONC 201. *Dose*: starting 250mg weekly, 3+3 dose escalation design. *Duration*: weekly intervals for up to 13 cycles (52 weeks)	Prophylactic Maintenance	Phase I	Adults age 19 or older with high risk AML or MDS who have undergone allo-HSCT 6-20 weeks prior to enrollment with adequate engraftment (ANC > 1000, PLT > 50).	20 (estimated enrollment)	DLT	Toxicities, Relapse, RFS	Not yet available
***MDM2 inhibitor***								
NCT05447663; Unpublished	Siremadlin (HDM201) ± DLI. S*tart*: between D+60 and D+120. *Dose*: 30-40 mg qD siremadlin capsules, orally (1-5 days of 28 or 42 days cycle)	Prophylactic Maintenance	Phase I/II	Adults age ≥18 years with high risk AML who have undergone allo-HSCT. Must not have active GVHD (acute or chronic) or history of grade 3 or 4 aGVHD.	8 (actual enrollment)	DLT	Relapse, GVHD, Pharmacokinetics	Not yet available
***Cellular/Immuno-Therapy/Modulator***								
NCT06197672; Unpublished	CD4-redirected chimeric antigen receptor engineered T-cells (CD4CAR)	Bridge to Transplant	Phase I	Adults age ≥ 18 with CD4+ recurrent or refractory AML.	30 (estimated enrollment)	MTD	Disease response	Not yet available
N/A; Yao et al. Frontiers in Oncology, 2019 (PMID: 31850234) (153)	Donor-derived CD123 CAR + TVFB RIC conditioning prior to Haplo-HSCT. *Dose*: 1.1 × 108 cells. *Schedule*: 1 day after preconditioning	Augmented Conditioning	Case Report	N/A	1	N/A	N/A	N/A
NCT00303667; Unpublished	Donor NK Cells. *Dose*: aldesleukin SQ 9 million units every other day beginning Day -12 through -2. NK Cells: Infusion on D-12; the targeted infused cell dose of CD3- CD19- selected NK product is within the range of 2-3 x 10^7 cells/kg	Peri-HSCT Cellular Therapy	Phase I/II	Adults age 18 to 70 years of age with high risk AML undergoing RIC haploidentical allo-HSCT.	50 (estimated enrollment)	6-month and 1-year DFS	GVHD, TRM, Incidence of post-transplant lymphoproliferative disorder	Not yet available
NCT03300492; Unpublished	Donor NK Cells. *Dose*: 1x10^7/kg, 1x10^8/kg and the remaining cells up to 1x10^9/kg. *Schedule*: expanded ex-vivo and infused on D+10, D+15, and D+20	Peri-HSCT Cellular Therapy	Phase I/II	Adults age ≥ 18 with AML or MDS without HLA-matched donor available deemed suitable for haploidentical allo-HSCT.	10 (estimated enrollment)	Incidence and severity of adverse events	1-year PFS. Morphologic and molecular remission rates. Incidence of graft rejection. NK cell doses given	Not yet available
NCT03927261; Unpublished	PRGN-3006 (autologous chimeric antigen receptor T cells)	Pre-emptive Therapy	Phase I/Ib	Adults age ≥ 18 years with MRD+ AML (or R/R AML, high risk MDS). Patients who are at least 3 months post allo-HSCT and/or 30-days post DLI are eligible. Patients with peripheral blasts >35% or CNS involvement are excluded.	88 (estimated enrollment)	DLT	Disease progression. Disease response in MDS patients. ANC recovery. ALC counts. Number of PRGN-3006 T Cells	
NCT06138587; Unpublished	Cytokine-Induced Memory-Like NK Cells. *Dose/Schedule*: Interleukin-2 1x daily every other day (7 doses total) on D+9, +11, +13, +15, +17, +19. D+7: Predetermined dose of CIML NK cells 1x daily.	Pre-emptive Therapy	Phase I	Adults age ≥ 18 years with AML, MDS, or MDS/MPN undergoing allo-HSCT at high risk for post-transplant relapse and MRD-positive. Patients with *FLT3*, *IDH*, or *BCR-ABL*-mutations excluded.	15 (estimated enrollment)	DLT	MTD, MRD-negativity rate, PFS, OS, Relapse, GVHD	Not yet available
NCT04623216; Unpublished: STIMULUS-AML2, ASH 2023 Oral Abstract 59	Sabatolimab (targets TIM3) ± Azacitidine. *Dose*: sabatolimab 400mg or 800mg IV every 4 weeks.	Prophylactic Maintenance	Phase Ib/II	Patients age 12 - 99 years of age with AML who are in CR with MRD-positivity after allo-HSCT anytime from D+100 to D+365.	59 (estimated enrollment)	DLT, % of patients without relapse	GVHD, MRD conversion	Not yet available
NCT05233618; Unpublished	Tagraxofusp. *Start*: between D+60 and D+120 days. *Dose*: escalating dose given D1-3 of cycles 1-4 of treatment (28 day cycles) and then on days 1 and 2 of subsequent cycles. *Duration*: up to 9 cycles	Prophylactic Maintenance	Phase I	Patients age 18 to 75 with higher risk CD123+ MF, CMML, or AML in remission pre-alloHSCT without GVHD.	44 (estimated enrollment)	DLT, Grade ≥3 AE, % of dose received	Time to relapse, death, or last contact GVHD	Not yet available
NCT04429191; Unpublished: ASH 2023 Oral Abstract 470	Briquilimab (JSP191), mAb against CD117 (c-Kit). *Dose*: 3 planned dose cohorts: 0.3 mg/kg, 0.6 mg/kg, and 1.0 mg/kg. When antibody has cleared, proceed to allo-HSCT	Conditioning Intensification	Phase I	Patients age ≥18 with AML or MDS with HLA-matched donor.	40 (estimated enrollment)	DLT, AE	N/A	Not yet available
NCT05823480; Unpublished	Magrolimab + Azacitidine	Prophylactic Maintenance	Phase I	Adults age 18 - 75 with high-risk MDS and AML scheduled to undergo allo-HSCT with adequate blood counts (ANC > 1500, PLT > 50,000).	44 (estimated enrollment)	RP2D	OS, PFS, CI-Relapse, NRM, GVHD, MRD	Not yet available
NCT02027064; Mo et al. Oncologist, 2018 (PMID: 30076280) (154)	IFN-alpha. *Start*: 3 months post allo-HSCT. *Dose*: 3 million units (for patients age > 16 years, and at 3 million units/m2 for those age < 16 years. *Duration*: 6 cycles (twice or thrice weekly of every 4 weeks cycle). *Median number of cycles*: 4.5	Pre-emptive Therapy	Phase IV	Patients age up to 65 years with high risk t(8;21) AML with molecular relapse after allo-HSCT without active GVHD.	42	1-year relapse rate: 7.2%	1‐year EFS: 76.0%. 1-year DFS: 92.4%. 1-year OS: 92.5%	1‐year CI of severe aGVHD: 7.1%. 1-year CI of severe cGVHD: 4.8%
NCT01433965; Pham et al. BMT, 2021 (PMID: 34471239) (155)	Lenalidomide. *Start*: 6-12 months after transplantation. *Dose*: 5-15mg in 3+3 design 21 days of a 28-day cycle. *Duration*: total of 6 cycles. Median treatment duration: 131 days. *Completed all planned cycles*: 50%	Prophylactic Maintenance	Phase I	Patients age 18-65 years with unfavorable AML or MDS who underwent allo-HSCT and were in morphologic CR 6-10 months prior to starting maintenance. Active grade 1 or higher GVHD was exclusionary.	16	MTD: 10mg	With median follow-up of 1222 days, 2 relapses	Grade 3-4 anemia: 6.3% (1/16). Grade 3-4 neutropenia: 25% (4/16). Grade 3-4 thrombocytopenia: 6.3% (1/16). aGVHD: 12.5% (2/16)

In contrast, Gao et al. found that combination rhG-CSF with minimal dose decitabine (5mg/m^2^ on days 1-5) was associated with far lower rates of cytopenias (8% grade 3/4 neutropenia, 15% grade 3/4 thrombocytopenia), but improved CIR (15% vs 38.3%, hazard ratio 0.32 (95% CI, 0.18 to 0.57; P <.01) as compared to no intervention in their phase II open-label, multicenter randomized trial (156). Whether this difference in relapse prevention and cytopenias between post-transplant maintenance with azacitidine and minimal dose decitabine + rhG-CSF relates to differences between the DNMTi, the rhG-CSF, or related to patient cohort or other trial related factors remains unknown. An intriguing possibility is that the addition of growth factor in the maintenance setting augments anti-leukemic activity of decitabine, with the salutatory incidental benefit of reduced myelosuppression. Interestingly, Gao et al. also noted increased NK, cytotoxic T-cell, and Tregs with G-CSF and minimal dose decitabine. Azacitidine has also been studied in combination with growth factor, specifically GM-CSF in combination with azacitidine in a phase II non-randomized study with 24-month RFS and OS of 47% and 57% respectively (144). There have been various other studies investigating decitabine and oral CC-486 in phase I/non-randomized settings (142, 146, 147). Additionally, there has been interest in combining hypomethylating agents with venetoclax (reviewed below), other small molecules, as well as DLI with or without other small molecules (145, 149, 157).

*GVL Considerations:* Goodyear and colleagues investigated the impact of azacitidine on T regulatory (Treg) cells as well as anti-tumor CD8+ T cells after alloHSCT. They found increased Tregs in the early post-transplant period (first three months), as well as preliminary evidence of T-cell response to tumor antigens (158). An analysis of the RICAZA trial further suggested a link between CD8+ T cell responses, azacitidine exposure, and relapse risk (141). Overall, the literature is mixed, with some studies suggesting augmentation of GVL effects (159, 160), while others suggest impairment of anti-leukemic activity (161, 162). It is likely that hypomethylating agents exert pleomorphic effects on residual recipient immune cells, the donor graft, and leukemic cells. Studies of decitabine with checkpoint inhibitors further suggest influence of the microenvironmental niche and cellular expression state-dependent clinical activity (163, 164). Further studies could deconvolute in more granular detail the effects of hypomethylating agents on various T cell and NK cell subsets, of particular interest in the context of multi-agent maintenance therapy. There are a number of prospective studies reporting the combination of DLI and DNMTi. Two of these report on the prophylactic use of DLI + DNMTi (145, 148) while others report this combination at the time of frank relapse after transplantation (165, 166).

Take-home message:

- Given the absence of convincing clinical trial data, there is currently no clear indication for single-agent DNMT inhibitors in subcutaneous or intravenous formulations in the prophylactic maintenance setting after alloHSCT (though pre-emptive use with rising MRD or dropping chimerism may be reasonable clinical scenarios).

### Venetoclax

The oral, selective BCL-2 inhibitor venetoclax offers another therapeutic opportunity with broad activity, with demonstration of activity against *NPM1*, *IDH1/2*, splicing mutations, and now secondary-ontogeny cases (57, 167). Venetoclax with azacitidine is a new first-line therapeutic option for patients with AML ineligible for intensive chemotherapy based on the VIALE-A trial and it has also been studied in combination with low-dose ara-C and IDH inhibitors in the up-front setting (56, 168, 169). In the transplant setting, venetoclax has been studied in combination with RIC and MAC chemotherapy regimens in patients with high-risk myeloid malignancies (65, 170). The VIALE-T study is recruiting patients to study venetoclax with azacitidine maintenance versus standard best supportive care after alloHSCT (NCT04161885). We have recently reported our phase I single-center experience with prophylactic venetoclax plus azacitidine maintenance after RIC-Ven conditioning in 22 patients with high risk MDS/AML. Cytopenias were the most common adverse event but transient, and infections were uncommon (2 COVID19 infections and 2 non-COVID19 infections; all grade 1-2) (143). After a median follow-up of 25 months, the 2-year OS, PFS, NRM, and CIR rates were 67%, 59%, 0%, and 41%, respectively. We did not observe any significant impact on T cell expansion but did note delayed CD19+ B cell reconstitution in patients receiving maintenance therapy compared to a historical cohort that did not receive maintenance. Persistent NGS-MRD was associated with relapse, and dynamic monitoring suggested that day 28 and day 100 timepoints may be useful in detecting incipient relapse. A randomized study will be needed to determine the value of venetoclax-based prophylactic maintenance therapy, but our study demonstrates reasonable safety and supports further investigation of pre-emptive azacitidine/venetoclax maintenance in high risk MDS/AML patients with persistent MRD early post-transplant. This study is now enrolling patients to a separate cohort to test the safety and efficacy of an all-oral post-transplant prophylactic maintenance regimen with combination decitabine/cedazuridine and venetoclax (NCT03613532).

*GVL considerations:* given the central role of apoptosis in T cell development, BH3 mimetics have been studied to interrogate their effects on the immune system. Outside of the transplant setting, studies of patients with AML and CLL exposed to venetoclax have found limited impact on T and NK cells (171). However there may be a rationale for apoptosis targeting in the transplant setting, as the combination of venetoclax has also been suggested to increase cytotoxic T cell activity/GVL via reactive-oxygen species generation augmented by DNMTi-mediated AML immunoreactivity via the STING-cGAS pathway (172). NK cells may also play a role in GVL, with data supporting targeting mitochondrial apoptosis to augment NK cell immunotherapy (173). Ludwig and colleagues studied prolonged (several month) venetoclax exposure in a mouse model of bone-marrow transplantation (174). They found no change in T-cell subset proportions but did identify lower T cell numbers and evidence of apoptotic adaptation. They also identified transcriptional and signaling pathway changes which may have effects on T cell function and implications for the GVL effect. Their work builds on findings from Kohlapp and colleagues suggesting venetoclax augments immune-checkpoint mediated anti-tumor T-cell activity, as well as prior work by Carrington and colleagues that categorized BH3 mimetics as immunomodulatory agents (175, 176).

Take-home message:

- Despite concern for persistent myelosuppression or increased infection risk, the current preliminary data suggest reduced doses of venetoclax-azacitidine combinations can be safely delivered in the post-transplant setting.
- Dynamic MRD testing may identify patients that are likely to benefit the most from venetoclax-azacitidine post-transplant maintenance.

## Novel/unapproved agents

### TP53:

Mutations in *TP53* portend dismal outcomes in AML. PRIMA-1 was identified in a screen of compounds inducing apoptosis in human cancer cell lines through restoration of p53 transcriptional function (177). APR-246 (an analog of PRIMA-1, also known as eprenetapopt) was first studied in humans in prostate cancer and refractory hematologic malignancies (178). Eprenetapopt was then studied in combination with Azacitidine in *TP53-*mutated MDS and AML (179, 180). It has also been studied in a phase I study in combination with azacitidine and venetoclax (181). In a phase II, multicenter open-label trial, eprenetapopt was combined with azacitidine as maintenance after alloHSCT in 33 patients with mutated *TP53* MDS or AML (150). Patients received azacitidine for 5 days and eprenetapopt for 4 days in each 28-day cycle. Patients received up to 12 cycles. The primary outcome was RFS and safety. Median RFS was 12.5 months and 1-year RFS was 59.9% with a 1-year OS of 78.8%. Despite the initial enthusiasm with this phase II study, to our knowledge, there are no active clinical trials studying eprenetapopt as post-alloHSCT maintenance in *TP53*-mutated MDS or AML.

### NPM1/KMT2A

Menin, a chromatin adaptor which interacts with MLL1/KMT2A and is implicated in the pathogenesis of *NPM1*-mutated AML, has been studied as a therapeutic target in both myeloid and lymphoid malignancies (182, 183). Menin inhibitors have been studied in early-phase clinical trials of *KMT2A*-rearranged or *NPM1*-mutated leukemia, with MEN1 mutations implicated in resistance mechanisms (184, 185). There is some data to suggest synergism with venetoclax (186). There are multiple phase I and II clinical trials studying menin inhibitors in combination with hypomethylating agents and conventional chemotherapy in patients with acute leukemias, including preliminary data in the post-transplant setting (NCT05360160, NCT05326516, NCT04067336, NCT04811560, NCT05453903, NCT05521087, NCT05153330). Issa and colleagues reported 3 patients treated with SNDX-5613 (revumenib) as post-transplantation maintenance in the AUGMENT-101 phase I trial, with long-term remissions in heavily pretreated patients (ASH 2022 Abstract #723). The on or off-target effect of menin inhibition on the donor immune system has not yet been elucidated.

### RAS-pathway

There is considerable interest in targeting the RAS-pathway given its role in leukemic pathogenesis and as a resistance pathway after FLT3-inhibitor, IDH1/2-inhibitor, and venetoclax exposure (167, 187–189). Studies have evaluated the MEK inhibitor trametinib in combination with azacitidine and venetoclax in R/R AML with RAS-pathway mutations or in combination with an AKT inhibitor (190, 191). Selumetinib and binimetinib are MEK inhibitors studied in the relapsed/refractory setting (192, 193). Oral AKT inhibitors have also been studied as single agents (194). To our knowledge, none of these agents are currently under investigation in the post-transplant setting.

### Other agents

Lenalidomide has been studied as post-alloHSCT maintenance in high-risk myeloid malignancies (155). A translational study of the HOVON-103 AML/SAKK30/10 study reported changes in microvascularization as well as T-cell mediated GVL (195). The immunomodulatory effect of lenalidomide after alloHSCT may not be limited to GVL, as there is data to suggest impact on T-cell trafficking to the gut with potential impact on GVHD (196). Other agents that have been studied after alloHSCT include pegylated IFN-2a (197) and the deacetylase inhibitor panobinostat (151).

Although data with mouse-double-minute-2 (MDM2) inhibitors in combination with chemotherapies or venetoclax have shown limited clinical activity in the setting of overt relapsed disease, renewed interest has been generated for its potential in the post-transplant setting based on preclinical data suggesting MDM2 inhibition increased TRAIL-receptor-1 and -2 MHC-II expression on leukemia cells (198, 199). These data prompted the development of a clinical trial (NCT05447663) to assess for safety and efficacy with post-transplant siremadlin (MDM2 inhibitor) prophylactic maintenance therapy with or without donor lymphocyte infusion in AML patients determined to be at high risk for relapse.

### Chimeric antigen receptor therapy

CAR-T cell therapy is now used routinely in the care of patients with B-cell hematologic malignancies, and there is increasing interest in developing CAR constructs for use in myeloid malignancies. Challenges in the myeloid space include the identification of antigens which can be targeted on leukemic clones while sparing healthy hematopoietic stem cells and precursors. Recent advances in target identification and selection are showing promise in realizing this specificity, and we refer readers to a recent review summarizing this rapidly evolving field (200, 201). Yao and colleagues report a case utilizing donor CD123 CAR-T as part of reduced intensity conditioning prior to haploidentical transplantation in a highly aggressive leukemia relapsing post allogeneic transplantation (153). Anti-CLL1 CAR-T therapy may serve as a bridge to alloHSCT, and has been studied in early phase trials in both children and adults with relapse/refractory disease (202, 203).

**Table 1** summarizes non-randomized trials of conditioning augmentation, preemptive maintenance, and prophylactic maintenance, while **Table 2** summarizes randomized clinical studies, both published and those registered at clinicaltrials.gov.

**Table 2:** Randomized trials of augmented conditioning and post-transplantation maintenance.

Trial ID (NCT or other)/Reference	Agent/Dose/Schedule	Strategy	Study Type	Patient Population (Key Inclusion/Exclusion)	N	Primary Outcome Intervention vs Control	Key Secondary Outcomes Intervention vs. Control	Key Toxicities Intervention vs. Control
***FLT3***								
EudraCT 2010-018539-16 DRKS00000591; SORMAIN: Burchert et al. JCO, 2020 (PMID: 32673171) (93)	Sorafenib vs Control/Placebo *Start*: D+60 to D+100 *Dose*: 200 qD -> 400mg BID *Duration*: 24 months or relapse/intolerability Median duration of sorafenib therapy: 34.57 weeks	Prophylactic Maintenance	Randomized Phase II	Adults with FLT3-ITD+ AML In CR after 9/10 or 10/10 matched allo-HSCT	83	RFS: 2y 85.0% vs 53.3% HR, 0.256 (P = .002)	2 year probability of survival: 90.5% vs 66.2% HR for death: 0.241 (P = 0.007)	Neutropenia: 2.4 vs 2.6% Infections: 26.2% vs 23.1% Thrombocytopenia: 4.8% vs 2.6% Overall GVHD: 76.8% vs 59.8%
NCT02474290; Xuan et al. Lancet Oncol. 2020 (PMID: 32791048) (94) Xuan et al. Lancet Haematol, 2023 (PMID: 37414062) (95)	Sorafenib vs Control/Placebo *Start*: D+30 up until D+60, median time to start 30 days *Dose*: 400mg BID *Duration*: until D+180 Median exposure to sorafenib: 134 days Completed maintenance at 400mg BID *Dose*: 38 patients	Prophylactic Maintenance	Randomized Phase III	Adults age 18 - 60 with FLT3-ITD+ AML in CR before and after allo-HSCT with hematologic recovery within 60 days of allo-HSCT and without active GVHD, infections, or kidney/liver dysfunction FYI: all received MAC allo-HSCT from matched or haplo-donors	202	1-year CI-relapse: 7.0% vs 24.5% HR 0.25 (p=0.0010)	OS: 72.0% vs 55.9% (HR 0.55, p=0.011.) LFS: 70.0% vs 49.0% (HR 0.47 p=0·0007) NRM (15·0% vs 14·7% (HR: 0·79, p=0.98)	Neutropenia: 9% vs 4% Infections: 25% vs 24% Thrombocytopenia: 13% vs 6% aGVHD: 23% vs 21% cGVHD: 18% vs 17%
NCT01883362; RADIUS: Maziarz et al. BMT, 100 (PMID: 33288862)	Midostaurin vs standard of care *Start*: D+28 to D+60 *Dose*: 50mg BID *Duration*: 12 4-week cycles, (~1 year) Completed protocol-specified therapy: 53%	Prophylactic Maintenance	Randomized Phase II	Adults age 18 - 70 with FLT3-ITD+ AML Allo-HSCT in CR1 Required heme-recovery, transfusion independence, controlled GVHD	60	18-month RFS: 89% vs 76% HR 0.46 (P = 0.27)	24-month RFS 85% vs 76% (HR, 0.60 [95% CI, 0.17–2.14]; P = 0.4297) 24-month OS 85% vs 76% (HR, 0.58 [95% CI, 0.19–1.79]; P = 0.34),	Anemia: 7% vs 10% Neutropenia: 7 vs 13% Febrile Neutropenia: 3% vs 7% aGVHD: 50% vs 53% cGVHD: 30% vs 33%
NCT02997202; Unpublished: BMT-CTN 1506 (MORPHO) Abstract LBA2711, EHA 2023	Gilteritinib vs Placebo *Start*: D+30 to D+90 (after engraftment) *Dose*: 120 mg/day *Duration*: 24-months	Prophylactic Maintenance	Randomized Phase II	Adults with FLT3-ITD+ AML in CR1 No more than 2 induction cycles	356	2-year RFS: 77.2% vs 69.9%. HR for RFS: 0.679; 95% CI, 0.459-1.005; P = .0518	OS: HR 0.846; 95% CI: 0.554, 1.293; 2-sided p- value: 0.4394 Gilteritinib effect MRD+ (HR=0.515, 95% CI: 0.316, 0.838, p = 0.0065) Gilteritinib effect in MRD- (HR=1.213, 95% CI: 0.616, 2.387, p = 0.575)	Neutropenia: 42.1% vs 15.8% cGVHD: 52.2% versus 42.1%
***DNMTi ± Small Molecular***								
NCT00887068; Oran et al. Blood Advances, 2020 (PMID: 33170934) (140)	Azacitidine vs Observation *Start*: enrolled between D+42 and D+100 (median time to C1, D+62) *Dose*: 32mg/m2/day SQ for 5 days every 28-day cycle *Duration*: 12 monthly cycles (median cycles received: 4) *Completed all planned cycles*: 27.6%	Prophylactic Maintenance	Randomized Phase III	Adults age 18 - 75 with AML and MDS with high risk features, induction failure, relapsed disease, or in CR2+ prior to allo-HSCT. Engrafted post allo-HSCT In morphologic CR by D+28 Adequate hepatic and renal function	187	Median RFS: 2.07 years vs 1.28 years (P = 0.43)	Median OS: 2.52 years vs 2.56 (P = 0.85) 1-year CI Relapse: 41% vs 39% 1-year transplant related mortality: 4.3% vs 5.3%	Grade 3+ Bone marrow suppression: 67% (58/87) vs 5.3% (5/94) 100 day: Grade 3-4 aGVHD: 4.3% vs 2.1% 1-year incidence of cGVHD 25.8% vs 30.8%
NCT04173533; Unpublished: AMADEUS	Azacitidine (oral CC-486) vs Placebo *Start*: Between D+42 and D+84 *Dose*: 200 mg once daily for first 14 days of each 28 day cycle *Duration*: up to 12 cycles	Prophylactic Maintenance	Randomized Phase III	Individuals age ≥16 with AML (in CR) or MDS (<10% blasts) undergoing allo-HSCT with engraftment within 14 days of starting treatment (ANC > 1000, PLT > 50,000) and with adequately controlled GVHD	324 (estimated enrollment)	12-month RFS: not published	Not published: OS, CI-Relapse, NRM, GVHD, Safety, QOL	Not yet available
NCT04161885; Unpublished: VIALE-T	Azacitidine+Venetoclax vs Best Supportive Care Azacitidine: daily on D1-5 of each 28-day cycle for up to 6 cycles Venetoclax: daily on D1-D28 for up to 24 cycles	Prophylactic Maintenance	Randomized Phase III	Patients age >18 (part 1) or age > 12 (part 2), with AML undergoing allo-HSCT, blasts <10% before allo-HSCT in CR after transplant. Patients with disease progression during prior treatment with venetoclax are excluded	424 (estimated enrollment)	DLT, OS	Not published: RFS, GVHD, MRD-response,	Not yet available
NCT05449899; Unpublished	Decitabine+GCSF+BuCy vs Decitabine+GCSF+FluBu G-CSF 5 ug/kg/day on D-17 to D-10 Decitabine 20mg/m2/day on D-14 to D-10	Augmented Conditioning	Randomized Phase II/III	Patients age 18 to 65 years with secondary AML undergoing allo-HSCT without therapy-related disease, prior allo-HSCT, uncontrolled infection, or other organ dysfunction	232 (estimated enrollment)	1-year NRM	1-year: OS, DFS, CIR as well as adverse effects	Not yet available
ChiCTR-IIR-16008182; Gao et al. JCO 2020 (PMID: 33108244) (156)	Decitabine + rhG-CSF vs Control In the interventional G-Dec group: rhG-CSF: 100 µg/m2 SQ on days D0-D5 Decitabine: 5 mg/m2 IV D1-D5 *Duration*: 6 courses between 6-8 weeks in length *Completed all planned courses*: 96%	Prophylactic Maintenance	Randomized Phase II	Patients with high risk AML with engrafted donor hematopoiesis in MRD-negative CR without GVHD and without uncontrolled infection	204	2-year CI-Relapse: 15.0% vs 38.3% HR of 0.32; P < .01	2-year CI of cGVHD without relapse: 23.0% vs 21.7% P = .82 2-year CI of TRM 3.4% vs 1.6% P = .44 2-year OS: 85.8% vs 69.7%, P = .01 2-year LFS: 81.9% vs 60.7%, P < .01	Grade 3+ Anemia: 18% vs 22.5% Grade 3+ Thrombocytopenia: 15% vs 9.8% Grade 3+ Neutropenia: 8% vs 6.9%
***Sphingosine-1-phosphate receptor***								
NCT05429632; Unpublished: MO-TRANS	Mocravimod vs Placebo *Dose*: 1mg or 3mg daily *Duration*: 12 months	Prophylactic Maintenance	Randomized Phase III	Adults age 18 - 75 years with ELN intermediate or high-risk AML CR1 (or any ELN risk in CR2) undergoing allo-HSCT	249 (estimated enrollment)	1-year RFS	2-year OS	Not yet available
***SMO Targeting***								
NCT04168502; Unpublished	Glasdegib vs Clinical Observation Maintenance with glasdegib 100 mg daily for one year or until toxicity/relapse	Prophylactic Maintenance	Randomized Phase III	Adults age 18 - 60 with untreated AML (excluding FLT3-mutated AML, tAML, secondary AML)	414 (estimated enrollment)	2.5-year DFS	N/A	Not yet available
***Cellular Therapy***								
NCT03597321; Unpublished: ELIT-AML01	DLI vs No Intervention	Prophylactic Maintenance	Randomized Phase II	Adults age 18 - 70 with AML in CR from non-cord donor allo-HSCT and without grade 2-4 aGVHD	124 (estimated enrollment)	2-year RFS	N/A	Not yet available

## Conclusion

AML is no longer a singular disease entity, but rather is an increasingly complex condition that is subdivided into genomic subgroups with distinct sensitivity to traditional chemotherapy and novel agents that target a growing number of drivers of disease pathogenesis. For higher risk myeloid malignancies, allogeneic stem cell transplantation remains the only curative treatment modality through the graft-vs-leukemia effect. However, transplantation is not universally effective, and despite an expanding armamentarium of targeted agents and immune-based therapies for the treatment of AML, post-transplant relapse remains a significant challenge with near universal poor outcomes. Prevention of post-transplantation relapse with prophylactic or pre-emptive maintenance therapy has the potential to decrease relapse risk and improve overall survival. Maintenance therapy must prove anti-leukemic efficacy, yet balance amelioration of relapse with treatment toxicity, effects on quality-of-life, time-toxicity, and the impact on the donor graft.

The value of MRD is becoming more evident in the post-transplant setting. It may serve as a dynamic marker identifying those patients who would most benefit from relapse prevention strategies, while sparing patients at lower relapse risk from treatment-related toxicity. Because AML presents as a clonal disease with genomic alterations detected in routine clinical care, serial molecular MRD assessments are a modality that could be broadly deployed, informing relapse risk, and identify new therapeutic vulnerabilities that may not have been apparent at diagnosis. However, the genomic heterogeneity of AML complicates the routine incorporation of NGS-MRD into clinical practice. When interpreting NGS-MRD, persistent clonality can reflect a spectrum of biologically diverse reservoirs of disease with the possibility of distinct relapse risk and kinetics profiles. This is in contrast to other hematologic malignancies, such as those driven by the t(9;22) translocation, in which a single genetic lesion can be more easily correlated with leukemic burden and relapse risk. Deconvoluting the gene/phenotype-specific subtypes of persistent clonality will necessitate large-scale, multi-institutional, programmatic efforts to develop standardized definitions of clinically meaningful NGS-MRD for incorporation into prospective randomized trials powered for patient-centered outcomes. Efforts are underway through the FNIH, collaborative projects such as pre-MEASURE and MEASURE, and the ELN MRD Working Party to build the evidence base necessary for clinical adoption of NGS- MRD in AML (28, 45).

While significant advances in our understanding of leukemogenesis has led to development of gene/mutation-specific targeted agents, our understanding of the long-term impact of these agents on the normal/graft immune system is quite limited. Hematopoietic reconstitution after alloHSCT is a complex process with implications for recovery of counts sufficient to conduct daily life, for immunity against a wide array of microbes, and for surveillance against non-hematologic malignancy. For these reasons, correlative studies of immune reconstitution and function should be incorporated into studies of post-alloHSCT maintenance therapy. Targeted agents are often complicated by cytopenias, suggesting an impact on normal hematopoiesis. Whether there is a concomitant clinically relevant impact not only on cell number, but cell function is largely unknown. As new agents are developed in the pre-clinical setting, efforts should be made to study any impact on normal hematopoiesis and on immune effector function. We envision these parallel avenues of investigation leading to targeted agents which harness a growing understanding of the genomic pathobiology of leukemia with ever more nuanced understanding of the GVL effect to reduce the risk of relapse and improve outcomes for patients afflicted with this often-devastating disease.

## Author contributions

HM: Conceptualization, Data curation, Formal analysis, Investigation, Methodology, Project administration, Validation, Writing – original draft, Writing – review & editing. VH: Investigation, Validation, Writing – review & editing. JG: Conceptualization, Data curation, Formal analysis, Funding acquisition, Investigation, Methodology, Project administration, Resources, Supervision, Validation, Visualization, Writing – original draft, Writing – review & editing.
